# A Multidisciplinary Approach to Addressing Barriers in Osteoporosis Screening, Diagnosis, Therapy Sequencing, and Adherence in India

**DOI:** 10.7759/cureus.110111

**Published:** 2026-06-02

**Authors:** Nitin Kapoor, Sundeep K Upadhyaya, Arvind G Kulkarni, Jervin Mano

**Affiliations:** 1 Endocrinology, Diabetes, and Metabolism, Christian Medical College and Hospital, Vellore, IND; 2 Rheumatology, Indraprastha Apollo Hospitals, New Delhi, IND; 3 Spine Surgery, Bombay Hospital and Medical Research Centre, Mumbai, IND; 4 Medical Affairs, Abbott Healthcare Pvt. Ltd., Mumbai, IND

**Keywords:** denosumab, india, interdisciplinary communication, osteoporosis diagnosis, osteoporosis therapy, primary healthcare, risk assessment

## Abstract

Osteoporosis is a significant public health problem in India. In India, osteoporosis has unique characteristics; an earlier onset of disease is seen because of the inherently low peak bone mass in Indian subjects compared with that in Western populations. This leads to a higher burden of osteoporotic fractures. At the same time, other contributing factors include genetic predisposition, poor calcium diets, limited sun exposure, and an overall higher prevalence of metabolic loading factors such as diabetes. Despite this, awareness and screening remain inadequate. Diagnosis is delayed because access to dual-energy X-ray absorptiometry is limited, and poor long-term adherence to treatment compounds the difficulties of primary and secondary prevention. Early screening using validated tools such as the Fracture Risk Assessment Tool, the Osteoporosis Self-Assessment Tool for Asians, or the Simple Calculated Osteoporosis Risk Estimation is essential, particularly in high-risk populations. Risk stratification and imaging are key to guiding individualized treatment with antiresorptive agents such as denosumab and/or anabolic agents like teriparatide. Real-world barriers, including financial constraints, lack of awareness, and the absence of coordinated care models such as fracture liaison services, further limit effective management. With this background of special circumstances in Indian settings, a three-level care model is proposed, featuring community-level screening by primary care teams, diagnosis and initial treatment by specialists, and complex-case management centers with integrated support from allied healthcare professionals and community health workers. Implementing this stratified, multidisciplinary protocol has the potential to bridge existing gaps in osteoporosis care.

## Introduction and background

Osteoporosis is a global health problem affecting more than 200 million people and is a leading cause of fragility fractures and premature mortality [1]. It is defined as a skeletal disorder characterized by compromised bone strength, resulting from alterations in bone density and quality that increase fracture risk [2]. Bone mineral density (BMD), expressed as a T-score (the number of standard deviations from the mean BMD of a young healthy reference population), is used for diagnostic classification. A T-score of −1.0 to −2.5 defines osteopenia, and a T-score below −2.5 defines osteoporosis, as per the World Health Organization, 1994 report. Peak bone mass, the maximum bone density attained in early adulthood, is a critical determinant of lifelong fracture risk; individuals who accrue lower peak bone mass have less skeletal reserve to lose before reaching osteoporotic thresholds [3]. Hip and vertebral fractures are associated with long-term morbidity and mortality. With populations aging worldwide, the burden of osteoporosis and fracture-related complications is projected to rise substantially. Several countries have implemented population-level strategies to address this challenge. Established fracture registries in Japan demonstrate the importance of nationwide fracture surveillance and real-world outcomes [4,5]. India currently lacks an equivalent registry, representing an evidence gap that limits epidemiological surveillance and policy-making in this area.i

A systematic review and meta-analysis of Indian studies reported a substantial burden of osteoporosis among postmenopausal women, with pooled prevalence estimates of 29% at both the lumbar spine and femoral neck [6]. Another large observational study of 31,238 adults in India reported an osteoporosis prevalence of 18.3% in the general population and up to 37% in individuals aged 60 years or older, with nearly one-third of postmenopausal women affected; however, as this was a single-institution cohort, these figures should be interpreted as indicative rather than nationally representative [7]. The Indian population typically attains lower peak bone mass due to a combination of genetic predisposition, low dietary calcium and vitamin D intake, and inadequate bone accrual during childhood and adolescence compared with their Western counterparts. Indian women exhibit lower BMD than non-Asian women [8], and a longer duration of menopause is an independent predictor of osteopenia [9]. Indian men experience a steep rise in osteoporotic fractures after 65 years, often earlier, compared to Western cohorts. One-year post-fracture mortality in India is reported at around 12.9% and may approach 30% in older adults with comorbidities [10]. The DeVOS (Delhi Vertebral Osteoporosis) study reported the prevalence of vertebral fractures in men and women aged 50 years and older as 18.8% and 17.1%, respectively [11].

Bone loss occurs when the balance between osteoclast-mediated resorption and osteoblast-mediated formation shifts in favor of resorption, a process accelerated by estrogen withdrawal at menopause and by the age-related decline in bone formation [12]. In the Indian context, this vulnerability is further compounded by genetic and environmental factors. Indian subjects attain lower peak bone mass during adolescence, partly due to chronically inadequate dietary intake of calcium and vitamin D at critical periods of bone accrual, compounded by reduced sunlight-mediated cutaneous vitamin D synthesis in those who are largely indoors [13]. Additionally, estrogen withdrawal at menopause and the age-related decline in bone formation further accelerate bone loss, resulting in a significant proportion of the Indian population crossing the osteoporotic threshold at an earlier chronological age than their Western counterparts.

Awareness of osteoporosis remains low in India. A cross-sectional study among postmenopausal women from Northern India found that 73% did not know about osteoporosis [14]. Diagnostic limitations further complicate early detection and monitoring of these conditions. Dual-energy X-ray absorptiometry (DXA), considered the gold standard, is not uniformly available for diagnosing osteoporosis across India [15]. At the healthcare provider level, delayed diagnosis, time constraints, and inadequate follow-up contribute to suboptimal care [16]. Long-term medication adherence is also a significant challenge, with many patients discontinuing osteoporosis therapy and follow-up assessments, thereby increasing the risk of recurrent fractures [8].

Addressing these challenges requires culturally appropriate, accessible, and sustainable strategies. To inform this narrative review and ensure clinical relevance to the Indian context, input was obtained through structured expert discussions involving multidisciplinary clinicians. These discussions aimed to contextualize existing evidence and provide practical perspectives on screening, diagnosis, treatment sequencing, and approaches to improve long-term adherence in osteoporosis care in India.

## Review

Methodology

This manuscript was developed using a structured plan that combined a targeted literature search with input from a multidisciplinary expert team on this literature. The literature search was conducted in PubMed and Google Scholar to identify publications on the prevalence, screening, diagnosis, treatment, adherence, and long-term management of osteoporosis in India. The PubMed search strategy used the terms ("osteoporosis"[MeSH] OR "bone mineral density"[MeSH]) AND ("India"[tiab] OR "Indian"[tiab]) AND ("screening"[tiab] OR "diagnosis"[tiab] OR "treatment"[tiab] OR "adherence"[tiab] OR "fracture"[tiab]), limited to English-language publications from January 2012 to December 2025. Relevant peer-reviewed studies, national guidelines, and real-world data were included, while animal studies and non-English publications were excluded. Titles and abstracts were independently screened by two authors (NK and JM); discordant inclusion decisions were resolved by discussion with a third author (SKU). Full-text review was performed for articles where relevance was uncertain from the abstract. Where high-quality India-specific randomized controlled trial evidence was unavailable, recommendations were informed by international guidelines and adapted to the Indian context.

Expert discussions were conducted across three structured regional sessions involving a total of 30 clinicians from Western, Northern, and Southern India. The multidisciplinary panel comprised specialists in orthopedic surgery (n=16), spine surgery (n=5), endocrinology (n=5), and rheumatology (n=4). Sessions were held in a structured round-table format using a predefined thematic framework, with a moderator facilitating the discussion. These consultations were advisory in nature and did not constitute formal qualitative research or original data collection. Where expert opinion diverged from published evidence, both perspectives have been represented in the text.

Screening

A key priority in addressing osteoporosis in India is the early identification of high-risk individuals through appropriate screening tailored to the local population. Screening rates remain low; cohort-level data from clinical practice in India suggest that only approximately 20% of at-risk patients are diagnosed with osteoporosis [17], although nationally representative screening data remain limited. Many individuals in India present late in the course of the disease due to low awareness and sociocultural barriers.

Identifying Individuals for Early Assessment

The Indian Society for Bone and Mineral Research currently recommends screening for women over 60 and men over 65 years of age, with lower thresholds for women to approximately 10 years after menopause [8]. In the presence of any of the risk factors or clinical warning signs listed below, screening should be performed earlier than the age-based thresholds [8].

A range of chronic medical conditions, endocrine disorders, genetic bone diseases, early menopause or ovarian insufficiency, and long-term use of medications such as glucocorticoids are established osteoporosis risk factors. Individuals with chronic diseases affecting bone metabolism require early evaluation [18,19], including those with chronic lower back or pelvic pain, poor nutrition, or visual impairment. A personal or parental history of fragility fractures, particularly those of the hip or spine, also warrants early screening [9]. In patients with diabetes mellitus, fracture risk may be high despite normal BMD, warranting vigilance for silent fractures and fall risk [18].

A BMI <17 kg/m² is associated with reduced peak bone mass, while a BMI >35 kg/m² may impair bone quality. Low body weight (<50 kg), common in Asian populations, is a consistent predictor of fractures. Lifestyle factors such as smoking, physical inactivity, and inadequate sunlight exposure further compromise bone health [9]. Table 1 presents the common clinical conditions and medication use associated with osteoporosis [20].

**Table 1:** Clinical conditions and medications associated with osteoporosis 25(OH)D: 25-hydroxy vitamin D, ART: antiretroviral therapy, BMD: bone mineral density, CKD: chronic kidney disease, DXA: dual-energy X-ray absorptiometry, GI: gastrointestinal, PTH: parathyroid hormone, TSH: thyroid-stimulating hormone [21]

		Mechanism	Screening
A.	Clinical conditions		
i.	Early menopause	Estrogen deficiency increases bone resorption	DXA in women with premature menopause
ii.	Type 2 diabetes mellitus	Reduced bone quality	Evaluate for vertebral deformities, chronic back pain
iii.	CKD	Disturbed mineral metabolism and secondary hyperparathyroidism	Assess calcium, phosphate, PTH, and vitamin D levels
iv.	Rheumatoid arthritis and chronic inflammatory disease	Cytokine-mediated bone resorption and steroid exposure	Screen for bone loss in early aggressive disease
v.	Hyperthyroidism/ hyperparathyroidism	Accelerated bone turnover	Routine TSH, calcium, and PTH in low BMD cases
vi.	Malabsorption syndromes (celiac, IBD)	Reduced calcium and vitamin D absorption	Screen with 25(OH)D, celiac antibodies, or GI history
vii.	Chronic liver disease and post-‍transplant states	Altered vitamin D metabolism, immunosuppressant effects	Assess liver function and vitamin D status
B.	Medications		
i.	Glucocorticoids (chronic use)	Decrease bone formation, increase resorption	Assess duration and dose; consider baseline DXA
ii.	Chemotherapy agents	Induces hypogonadism, marrow toxicity	Post-treatment bone health evaluation
iii.	Immunosuppressants (cyclosporine and tacrolimus)	Increase resorption and reduce formation	Monitor transplant recipients
iv.	Anticonvulsants (phenytoin, carbamazepine, and valproate)	Increase vitamin D catabolism	Check vitamin D and calcium periodically
v.	Antiretrovirals (tenofovir disoproxil fumarate)	Direct osteoblast toxicity	Screen at ART initiation and annually
vi.	Anticoagulants (warfarin and heparin)	Impair vitamin K-dependent bone protein synthesis	Review long-term therapy (>6 months)
vii.	Chelating agents (in thalassemia)	Mineral deficiency, hypogonadism	Annual bone assessment
viii.	Antipsychotics (lithium and risperidone)	Hyperprolactinemia affects bone remodeling	Monitor prolactin levels
ix.	Proton pump inhibitors	Reduce calcium absorption	Evaluate chronic users (>1 year)
x.	Anti-tubercular drugs (isoniazid)	Pyridoxine deficiency affects collagen cross-linking	Evaluate for long-term users

Approaches to Osteoporosis Screening

An effective screening strategy for osteoporosis requires a combination of clinical evaluation, imaging modalities, and risk assessment tools to identify individuals at risk and guide timely intervention.

Clinical assessment: A baseline biochemical evaluation, including serum calcium, vitamin D, and parathyroid hormone levels, is essential, particularly in individuals with established risk factors, chronic disorders, or long-term medications known to impair bone metabolism [19].

Imaging: In standard settings, DXA scans or quantitative computed tomography (qCT) may be recommended for patients with a history of fragility fractures. According to the American Society for Bone and Mineral Research, the lowest T-score across measured sites should be used to assess skeletal fragility, as it best correlates with overall fracture risk [22]. Forearm BMD is particularly informative in primary hyperparathyroidism [23], while lumbar spine BMD is relevant in postmenopausal women due to early trabecular bone loss in this region. Conventional X-rays of the spine, femur, or jaw may be considered when advanced imaging is unavailable [8], although their diagnostic value is limited [24].

Risk assessment tools: When access to imaging is limited, clinical risk prediction models can serve as valuable alternatives. The Fracture Risk Assessment Tool (FRAX®), Osteoporosis Self-Assessment Tool for Asians (OSTA), Simple Calculated Osteoporosis Risk Estimation (SCORE), and Male Osteoporosis Risk Estimation Score (MORES) allow for practical, cost-effective estimation of fracture risk without requiring BMD input and are especially relevant in primary care and rural or underserved settings [25].

In postmenopausal women, FRAX® effectively predicts fragility fractures and can be used with or without BMD or trabecular bone score to guide clinical decisions, especially in India, where population-specific validation studies have confirmed its acceptable predictive utility [26]. Cost-effectiveness modeling studies suggest that starting FRAX®-guided treatment at age 50 years and universal treatment for individuals aged 65 years and older are efficient strategies to combat osteoporosis in India. The SCORE tool is similarly useful for identifying individuals at risk of femoral neck osteoporosis [27].

Peripheral ultrasound: Although peripheral ultrasound has been explored as a low-cost screening option, its diagnostic accuracy is limited by high false-negative rates [8].

The panel emphasized that the utility of DXA scans is limited by availability and cost in India. Screening decisions should be guided by clinical risk factors, including age, sex, steroid use, chronic illnesses, and fracture history. Table 2 presents an overview of the various screening tools [8,19,26-31].

**Table 2:** Overview of screening tools in osteoporosis BMD: bone mineral density, DXA: dual-energy X-ray absorptiometry, FRAX®: Fracture Risk Assessment Tool, MORES: Major Osteoporosis Risk Estimation Score, OSTA: Osteoporosis Self-Assessment Tool for Asians, qCT: quantitative computed tomography, QUS: quantitative ultrasound, SCORE: Simple Calculated Osteoporosis Risk Estimation [8,19,26-31]

Tool	Principle	Pros	Cons	Ideal use situation
DXA	Measures BMD at the spine, hip, and forearm	Gold standard; precise; monitors treatment; vertebral fracture assessment	Limited availability; cost; machine variability; technician-dependent	Standard screening in postmenopausal women, men >65 years, high-risk patients; follow-up every 1-2 years
qCT	3D volumetric BMD	Accurate; less affected by degenerative changes	Higher radiation, costly, and less available	Patients with spine/hip deformities or inaccurate DXA
FRAX®	10-year fracture risk using clinical factors ± BMD	No BMD required; validated in India; cost-effective	Does not account for falls, cumulative steroid exposure, or vertebral fractures	Primary care screening; rural/low-resource areas; referral guidance
OSTA	Age and body weight	Simple, low cost	Less precise; population-specific	Initial risk stratification in postmenopausal Asian women
SCORE	Clinical risk factors	Simple; practical; cost-effective	Limited predictive power without BMD	Resource-limited settings; primary care triage
MORES	Clinical risk factors in men	Simple, low cost	Less accurate than DXA; limited validation	Early identification of at-risk men in primary care
X-ray	Detects osteopenia or vertebral fractures	Widely available; low cost	Detects only after ~30% bone loss; low sensitivity	Preliminary assessment or incidental findings in resource-‍limited settings
QUS	Bone properties at peripheral sites	Low cost; portable; no radiation	Poor accuracy; high false negatives	Temporary screening where DXA is unavailable; community outreach

Barriers to Screening in Real-World Practice

**Figure 1:**
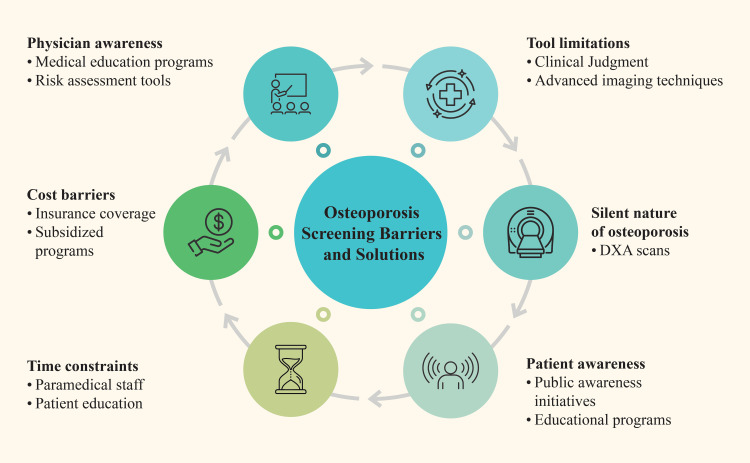
Barriers and solutions to osteoporosis screening DXA: dual-energy X-ray absorptiometry Image Credit: Authors using Adobe Illustrator version 30.0 (Adobe Inc., San Jose, CA)

The disease often progresses silently until fractures occur [32,33]. Osteoporosis screening remains underutilized in India due to multiple interacting barriers, as illustrated in Figure 1. DXA, the diagnostic gold standard, is largely concentrated in urban tertiary care centers, with limited reach in rural and tier-2/3 regions. QUS and conventional radiography offer more accessible alternatives in resource-constrained settings, albeit with diagnostic limitations [15,17,34]. Clinical risk tools such as FRAX®, OSTA, SCORE, and MORES enable cost-effective risk stratification at the primary care level without specialized infrastructure [29-31]. However, suboptimal awareness among both patients and physicians remains a significant gap [14,17].

Diagnosis

The diagnosis of osteoporosis extends beyond BMD alone and requires the integration of clinical examination, imaging, biochemical evaluation, and fracture risk prediction [8].

Clinical History and Examination

A detailed clinical evaluation forms the cornerstone of osteoporosis diagnosis, including a personal and family history of fragility fractures. A comprehensive assessment of comorbidities and medications, along with lifestyle factors, including smoking, alcohol use, diet, and physical activity, should be conducted. Clinicians should also assess mobility and fall risk [9,10,35].

Integrated Risk Assessment

While BMD assessment using DXA remains the standard for diagnosing osteoporosis, it alone does not accurately reflect overall fracture risk, particularly when osteopenia is present on the DXA scan. Combining risk assessment tools with clinical evaluation, vertebral fracture assessment findings, and fracture history enables risk stratification and individualized treatment. When results are discordant, treatment should be guided by the patient’s overall clinical risk [8]. In resource-limited settings, risk prediction tools such as FRAX® without BMD serve as an alternative. In select clinical scenarios, computed tomography-based assessments can complement DXA.

A routine biochemical evaluation to exclude secondary causes, along with renal and liver function tests, is advised. Complete blood counts, diabetes evaluation, and thyroid function tests should be obtained. Additional investigations are guided by clinical suspicion.

Bone turnover markers (BTMs) may be used selectively to evaluate treatment response and identify contributors to suboptimal outcomes. Serum CTX and procollagen type I N-terminal propeptide are recommended as reference BTMs by the International Osteoporosis Foundation [36]. They are most clinically useful in three specific scenarios in the Indian context: (1) early assessment of treatment response, with a meaningful reduction in CTX at three to six months expected with antiresorptive therapy; specific percentage thresholds vary by assay and have not been universally standardized; (2) identification of poor adherence, persistently elevated or rising BTMs during therapy should prompt a structured adherence review before escalating treatment; and (3) distinguishing treatment failure from non-adherence in patients with new fractures on therapy, BTM levels can help differentiate true pharmacological failure (BTMs appropriately suppressed) from non-adherence (BTMs not suppressed). While the Indian Rheumatology Association guidelines recommend BTMs alongside BMD for high-risk identification and monitoring, their clinical adoption remains limited [37].

Special Populations

Z-scores may aid interpretation in adults under 50 years and those over 70 years [8]. Chronic kidney disease (CKD), mineral, and bone disorders necessitate additional biochemical markers [21].

**Figure 2:**
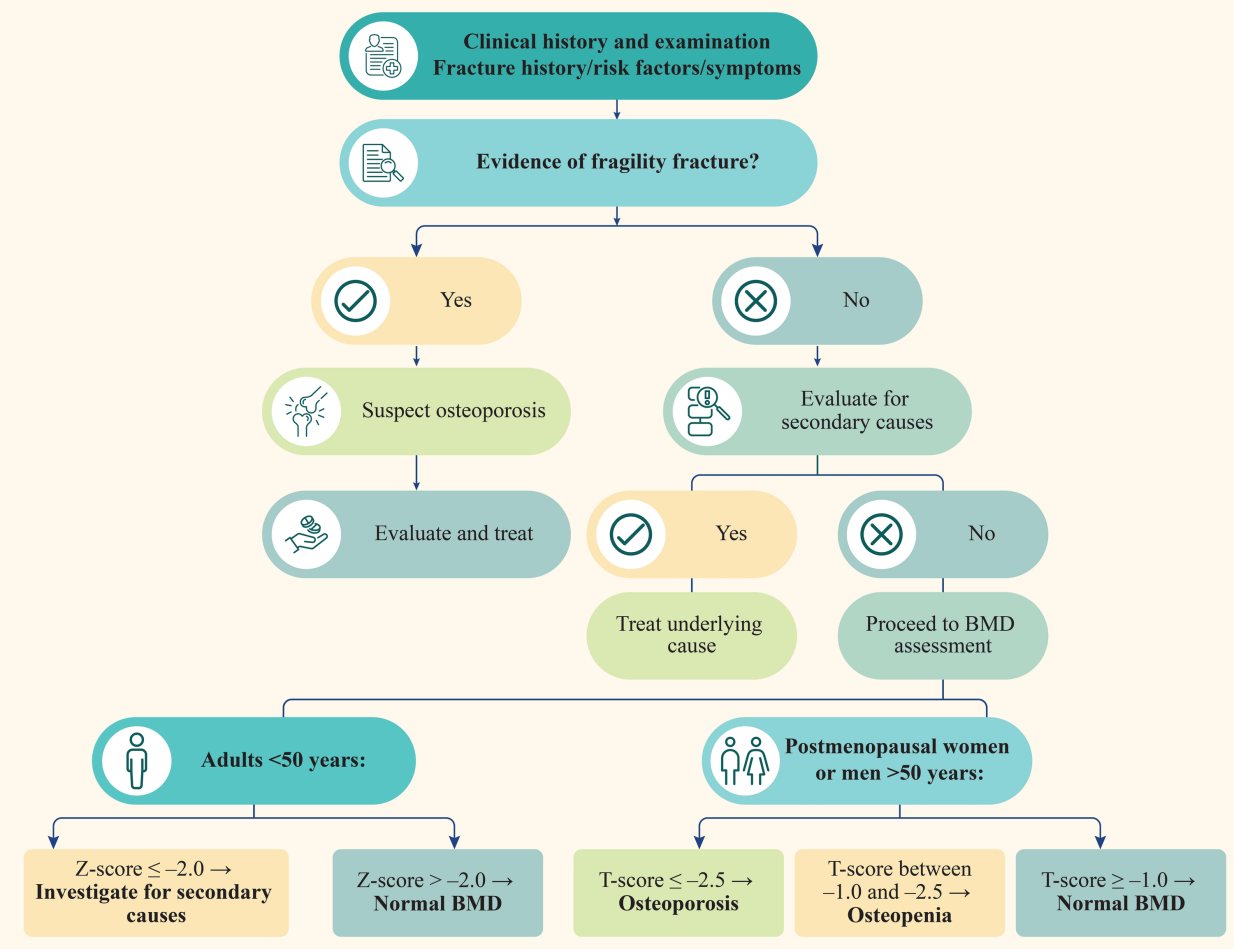
Osteoporosis diagnosis and risk management BMD: bone mineral density Image Credit: Authors using Adobe Illustrator version 30.0 (Adobe Inc., San Jose, CA)

Screening refers to the identification of individuals who warrant formal BMD assessment, based on age, sex, and clinical risk factors, as outlined in Table 1 and the Screening section of this manuscript. Diagnosis, in contrast, relies on interpretation of BMD using T-scores (for postmenopausal women and men over 50 years) or Z-scores (for adults under 50 years), as categorized by World Health Organization criteria and illustrated in Figure 2. These two processes are sequential and complementary; a positive screen triggers diagnostic workup, which in turn informs treatment decisions. The proposed algorithm for osteoporosis risk classification, which integrates fracture history, secondary causes, and BMD/FRAX® assessment, is illustrated in Figure 2.

Treatment

Effective management of osteoporosis requires a balanced approach with timely intervention and individualized risk assessment. This section outlines the core principles of treatment.

When to Start Therapy

Therapy should be considered for individuals at moderate to high fracture risk, including those with previous fragility fractures, low BMD, or additional clinical risk factors.

Identifying patients at very high fracture risk is a critical step for treatment decisions. Recent fragility fractures carry the highest short-term risk of a subsequent event. Patients sustaining multiple fragility fractures, irrespective of when they occurred, or those with fractures despite adherence to approved osteoporosis therapy, should similarly be regarded as very high risk, since these presentations indicate either skeletal fragility or inadequate therapeutic response [38].

Quantitative fracture probability, as estimated by the FRAX® tool, may complement clinical assessment; globally, thresholds of ≥20% for major osteoporotic fracture and ≥3% for hip fracture over 10 years have been proposed to guide intervention [38]. However, these cutoffs are derived from North American and European normative datasets and have not been prospectively validated in Indian populations. In the absence of India-specific FRAX® intervention thresholds, FRAX® estimates should be interpreted alongside clinical risk factors and BMD data [8].

Special Populations

Special considerations are required for specific patient groups, including younger adults with chronic illnesses, older adults with frailty, and those with secondary causes of osteoporosis. In these populations, treatment selection and timing should take into account factors such as renal function, fall risk, and long-term safety and be guided by structured monitoring protocols.

Principles of Therapy

Osteoporosis treatment should be goal-directed and personalized, with therapy guided by individual fracture risk assessment, imaging to detect silent vertebral fractures, and evaluation of modifiable risk factors. Baseline BMD measurement at the total hip, femoral neck, and lumbar spine is central to establishing treatment targets and monitoring therapeutic response [38].

Choice of Therapy

The selection of osteoporosis therapy should reflect the patient’s overall fracture risk profile and integrate both pharmacological and non-pharmacological strategies.

Non-pharmacological management and fall prevention

Non-pharmacological strategies are the cornerstone of osteoporosis management in India and are applicable across all risk categories, particularly given the high burden of vitamin D deficiency and underdiagnosis. Exercise interventions involving weight-bearing and resistance training improve BMD and reduce fracture risk when performed regularly. Multicomponent balance and strength training programs are effective in reducing falls in older adults [39]. In frail or post-fracture patients, supervised physiotherapy focusing on gait, balance, and strength is preferred. At the same time, culturally familiar practices such as yoga may offer feasible adjunctive options in community and primary care settings.

Nutritional measures remain a practical and low-cost component of care. Adequate calcium intake through diet is essential for skeletal health, while vitamin D deficiency is highly prevalent across India and contributes significantly to the risk of osteoporosis. In routine practice, supplementation is often required and can be guided by clinical assessment and availability [13].

These interventions are well-suited to primary care delivery. Community health workers can support early identification of individuals at risk and reinforce lifestyle and adherence counseling during household visits. Integration of bone health awareness and fall prevention strategies into existing national programs, such as the National Program for Non-Communicable Diseases, provides a scalable and sustainable implementation pathway in India.

Pharmacological management

Evidence from landmark randomized controlled trials has established the efficacy of both antiresorptive and anabolic therapies in reducing osteoporotic fractures. The Fracture Intervention Trial [40] demonstrated that in high-risk postmenopausal women, alendronate reduced vertebral fracture risk by approximately 47% and hip fracture risk by approximately 50%, cementing the role of bisphosphonates as the first-line therapy. The HORIZON [41] trial extended this evidence to intravenous zoledronic acid, which achieved reductions of approximately 70% in vertebral and 41% in hip fractures with annual infusions. Notably, it demonstrated a survival benefit following hip fracture. For the RANK-L inhibitor denosumab, the FREEDOM [42] trial demonstrated reductions of approximately 68% in vertebral, 40% in hip, and 20% in nonvertebral fractures, with a particularly favorable profile in patients with renal impairment, for whom bisphosphonates are contraindicated. The Fracture Prevention Trial established teriparatide's anabolic mechanism as mechanistically distinct, demonstrating reductions in vertebral and nonvertebral fractures of approximately 65% and 53%, respectively [43]. Finally, the DATA [44] trial demonstrated that combined teriparatide and denosumab therapy produced superior BMD gains at the spine and hip compared with either agent alone, providing the strongest evidence to date for combination anabolic-antiresorptive therapy in postmenopausal osteoporosis [44]. More recently, romosozumab, a sclerostin inhibitor with dual actions of increasing bone formation and reducing bone resorption, demonstrated significant reductions in vertebral and clinical fractures in the ARCH and FRAME trials [45]; however, its real-world uptake in India remains limited owing to its high cost and restricted availability [34].

Treatment duration and drug holidays

For oral bisphosphonates (alendronate, risedronate), treatment for three to five years is appropriate in patients at moderate risk, after which a drug holiday may be considered in those whose hip BMD T-score is above −2.5 and who have not sustained a hip or vertebral fracture [46]. For zoledronic acid, a holiday after three to six infusions is reasonable in similar low-risk patients [41]. Drug holidays are not recommended for patients at high or very high fracture risk. Drug holidays for two to three years can be considered for patients not at high risk [46]. Denosumab should not be discontinued abruptly due to the risk of rebound vertebral fractures; transition to bisphosphonates is recommended upon cessation [47]. Teriparatide is approved for a maximum of 24 months, after which an antiresorptive therapy must be initiated to preserve BMD gains [8].

Adverse Effects

**Figure 3:**
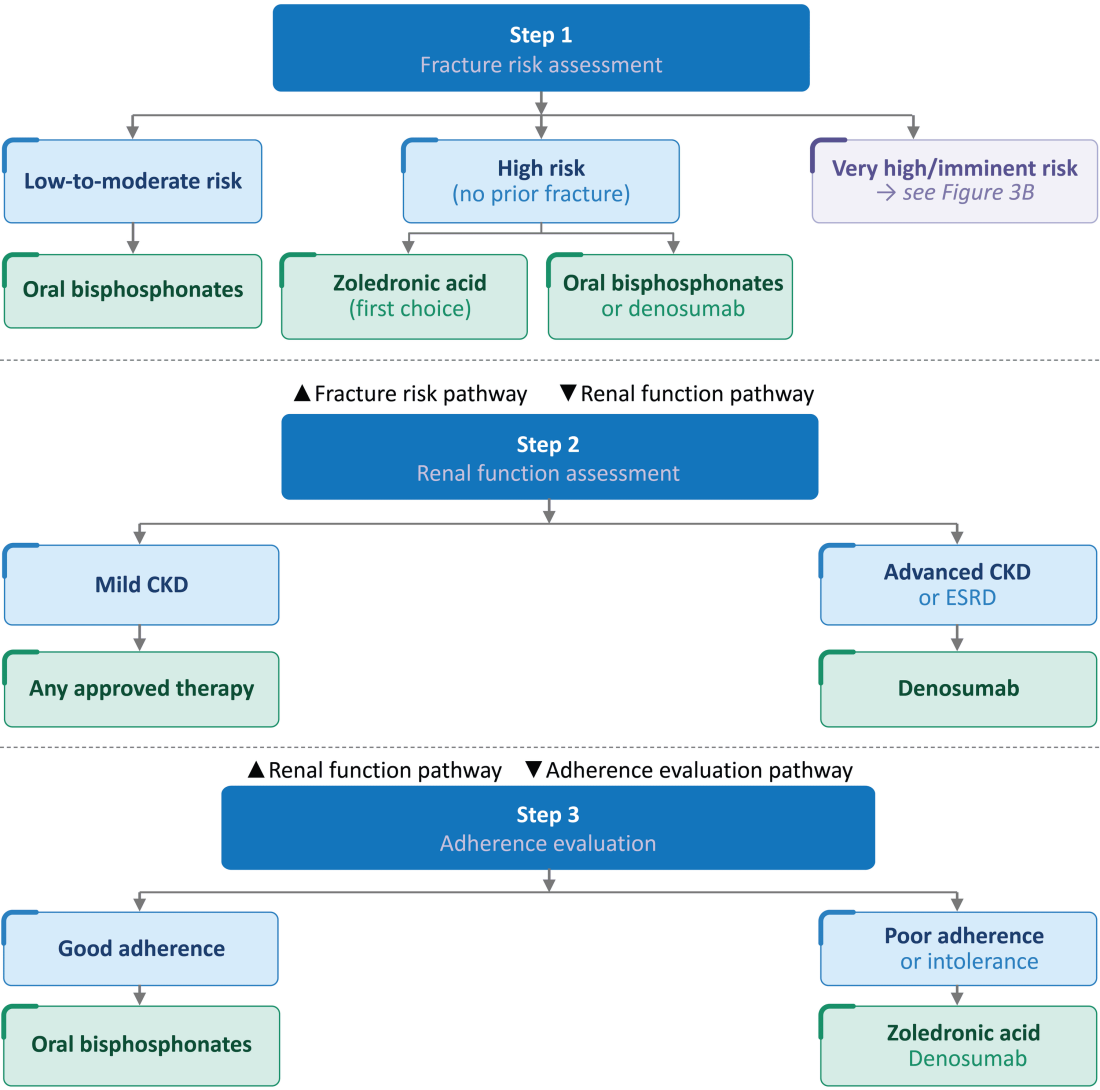
Antiresorptive therapy selection pathway CKD: chronic kidney disease, ESRD: end-stage renal disease Image Credit: Authors using Adobe Illustrator version 30.0 (Adobe Inc., San Jose, CA)

**Figure 4:**
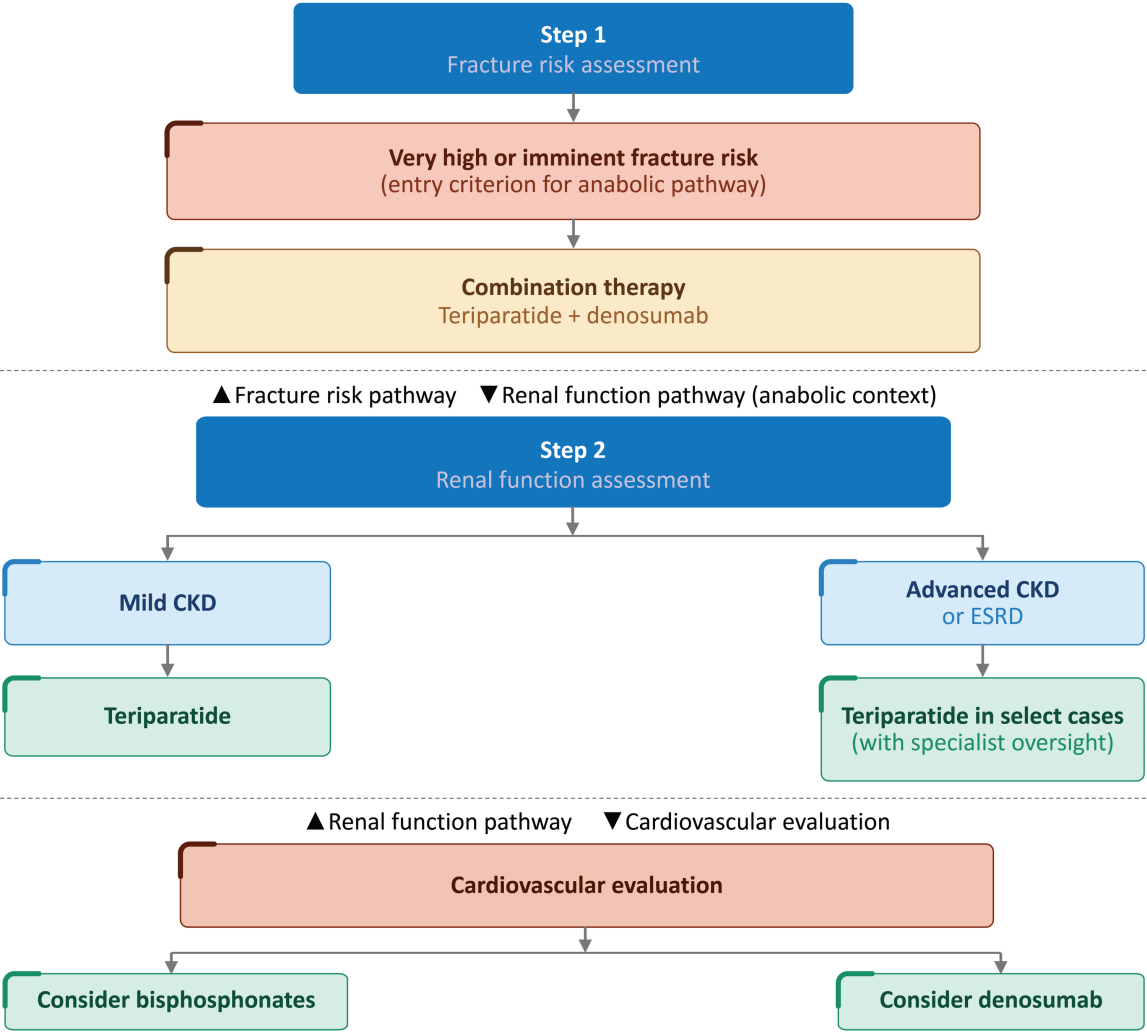
Anabolic and combination therapy selection pathway CKD: chronic kidney disease, ESRD: end-stage renal disease Image Credit: Authors using Adobe Illustrator version 30.0 (Adobe Inc., San Jose, CA)

Oral bisphosphonates may cause upper gastrointestinal intolerance, a recognized cause of discontinuation, and weekly or monthly formulations may improve adherence. Rare but serious complications include osteonecrosis of the jaw and atypical femoral fractures with prolonged use. Zoledronic acid may precipitate acute-phase reactions, generally manageable with symptomatic treatment. Denosumab may cause hypocalcemia, particularly in the context of vitamin D deficiency, which is highly prevalent in India, making calcium and vitamin D adequacy before initiation essential. Teriparatide carries a risk of hypercalcemia and is contraindicated in patients with prior skeletal radiation, Paget's disease, or elevated alkaline phosphatase [19]. Figure 3 and Figure 4 summarize the key factors to consider when selecting therapies for the management of osteoporosis.

Clinical modifiers, including glucocorticoid use, fracture history, and BTMs, may further influence the selection of anabolic therapy.

Cost and Access to Drugs in India

Cost and access barriers remain critical obstacles to osteoporosis management in India. DXA scanning costs USD 25-50 with minimal reimbursement, and over 60% of healthcare is delivered in the private sector, where outpatient osteoporosis medications are typically an out-of-pocket expense. Approximately 40% of the population has some form of health insurance, but coverage is often limited in scope. As shown in Table 1, bisphosphonates, denosumab, and teriparatide are available and reimbursed. However, reimbursement is largely confined to public hospitals and infrequent in the private sector, where most patients are managed [34]. Beyond initial treatment selection, appropriate sequencing and switching strategies are important for patients with a suboptimal response or an ongoing high risk of fractures.

Sequencing and Switching Therapy

For patients who do not achieve treatment goals or who experience new fractures while on therapy, sequencing or switching may be necessary. Strategies include transitioning from antiresorptive to anabolic therapy, combining agents in select cases, or cycling treatments based on BMD response and fracture risk reduction.

For patients at very high or imminent fracture risk, anabolic therapy with teriparatide is the preferred initial approach. The VERO trial demonstrated significantly lower rates of vertebral and clinical fractures with teriparatide versus risedronate in severe osteoporosis, supporting its use over antiresorptive therapy in this setting [48]. Combination therapy with teriparatide and denosumab may be considered in selected patients requiring maximal BMD gains [44]. Given cost and limited availability in India, this approach should be restricted to specialist settings; sequential therapy remains appropriate for most very high-risk patients [8,38].

Challenges to Treatment

Care delivery and system-level limitations further hinder long-term management. The absence of a national fracture registry limits monitoring of fracture burden and treatment patterns [4,5]. Persistent deficiencies in osteoporosis care include low rates of post-fracture evaluation, delayed initiation of appropriate therapy, poor long-term adherence, and weak coordination between specialists [8]. A fracture liaison service (FLS) provides the most effective coordinated pathway for managing patients after fragility fractures [49]. An FLS is usually led by a trained advanced practice provider and offers a cost-effective alternative to usual care, especially for older adults. Its core components include case identification, osteoporosis assessment, timely initiation of treatment, and fall prevention [49]. Despite this, FLS penetration in India remains critically low, with only 1-9% of hospitals operating an FLS and just 1% of fragility fractures identified or referred through such services [34].

Barriers and strategies for treatment adherence in real-world practice: Adherence to osteoporosis therapy remains a major clinical challenge in India. Cohort-level data from India indicate one-year persistence rates of approximately 64% [17], whereas international estimates vary widely across drug classes, dosing frequencies, and healthcare settings.

The delayed and often imperceptible benefits of treatment, together with the asymptomatic nature of osteoporosis, reduce patient motivation to continue therapy. Delays in referral from primary care and rural settings lead to missed opportunities for timely management. System-level barriers, including the lack of coordinated models, result in fragmented care. Additionally, medication costs and the long-term commitment required for therapy remain other deterrents [36].

Multidisciplinary care pathways, including FLS where available, can improve continuity of care and support secondary prevention. Policy-level interventions, such as government-supported food fortification initiatives and hospital-based screening protocols, can help identify and protect individuals at risk. Patient engagement tools, such as automated reminders, educational initiatives, and pharmacist-led interventions, may help reduce non-adherence rates.

Monitoring

Osteoporosis monitoring strategies should be tailored to the clinical context, the availability of tools, and the individual patient's needs.

Selection of Monitoring Tools

Close follow-up with BMD assessment and fracture surveillance is essential for guiding long-term management and evaluating therapeutic response. Monitoring should be guided by pre-specified therapeutic targets, with treatment goals including BMD improvement or stabilization, prevention of new fractures, and reduction of imminent fracture risk. In standard care settings, baseline DXA with repeat testing at one to two years is appropriate to assess treatment response; once BMD values stabilize, the interval may be extended to two to three years [50]. qCT can be used selectively when DXA is inconclusive or site-specific assessment is needed, with similar monitoring intervals [28]. BTMs may complement BMD monitoring by providing earlier indications of treatment response, although their use is limited by cost and availability. Physicians have limited awareness regarding their clinical utility [37]. Practical challenges include a lack of assay standardization and the absence of universally accepted or population-specific reference ranges [37]. In primary care or low-resource settings, especially in India, where more basic tools are often necessary, plain radiography may be used to confirm suspected fractures or to calculate FRAX® without BMD [31]. Table 3 presents various monitoring tools for osteoporosis.

**Table 3:** Monitoring tools in osteoporosis BMD: bone mineral density, BTM: bone turnover marker, DXA: dual-energy X-ray absorptiometry [26,28,31,37]

Tool	Usage	Frequency	Outcome
DXA (lumbar spine, total hip, and femoral neck)	Measure BMD to guide therapy	Every 1-2 years, depending on risk and treatment response	Stabilization or improvement in BMD, guiding therapy adjustments
Vertebral fracture assessment/spinal radiographs	Detect new or progressive vertebral fractures	As clinically indicated, when the risk profile changes	Early identification of fractures to optimize management
Laboratory tests (calcium, vitamin D, and BTMs)	Monitor adherence, secondary causes, and metabolic complications	Selectively, based on clinical context	Identify causes of suboptimal response and guide therapy modification
BTMs	Assess treatment response, evaluate adherence, and detect early changes in bone metabolism	At baseline and 3-6 months after therapy initiation or change	Indication of therapeutic effectiveness or nonresponse

Therapeutic Adjustments After Follow-Up

Once the monitoring strategy is selected, the next crucial step is to interpret follow-up findings to inform treatment decisions. Changes in BMD, fracture occurrence, and risk reclassification play a pivotal role in this process. Table 4 summarizes potential clinical scenarios after the first follow-up and the corresponding therapeutic actions.

**Table 4:** Follow-up outcomes and management strategies in osteoporosis * Reduction in BMD during treatment BMD: bone mineral density, BTM: bone turnover marker, FRAX®: Fracture Risk Assessment Tool

Follow-up outcome	Management approach
Improvement in BMD	Continue current drug therapy. In patients on bisphosphonates, consider a drug holiday if appropriate.
No significant change	Assess drug compliance and continue therapy or consider combination therapy. BTMs can be used in select cases. Escalate treatment if there is a new fracture, a higher FRAX^®^ score, or a shift to a higher risk category.
Failure of treatment*	Step up therapy to a more intensive regimen.

Role-Based Algorithm for Osteoporosis Management Across Healthcare Levels

**Figure 5:**
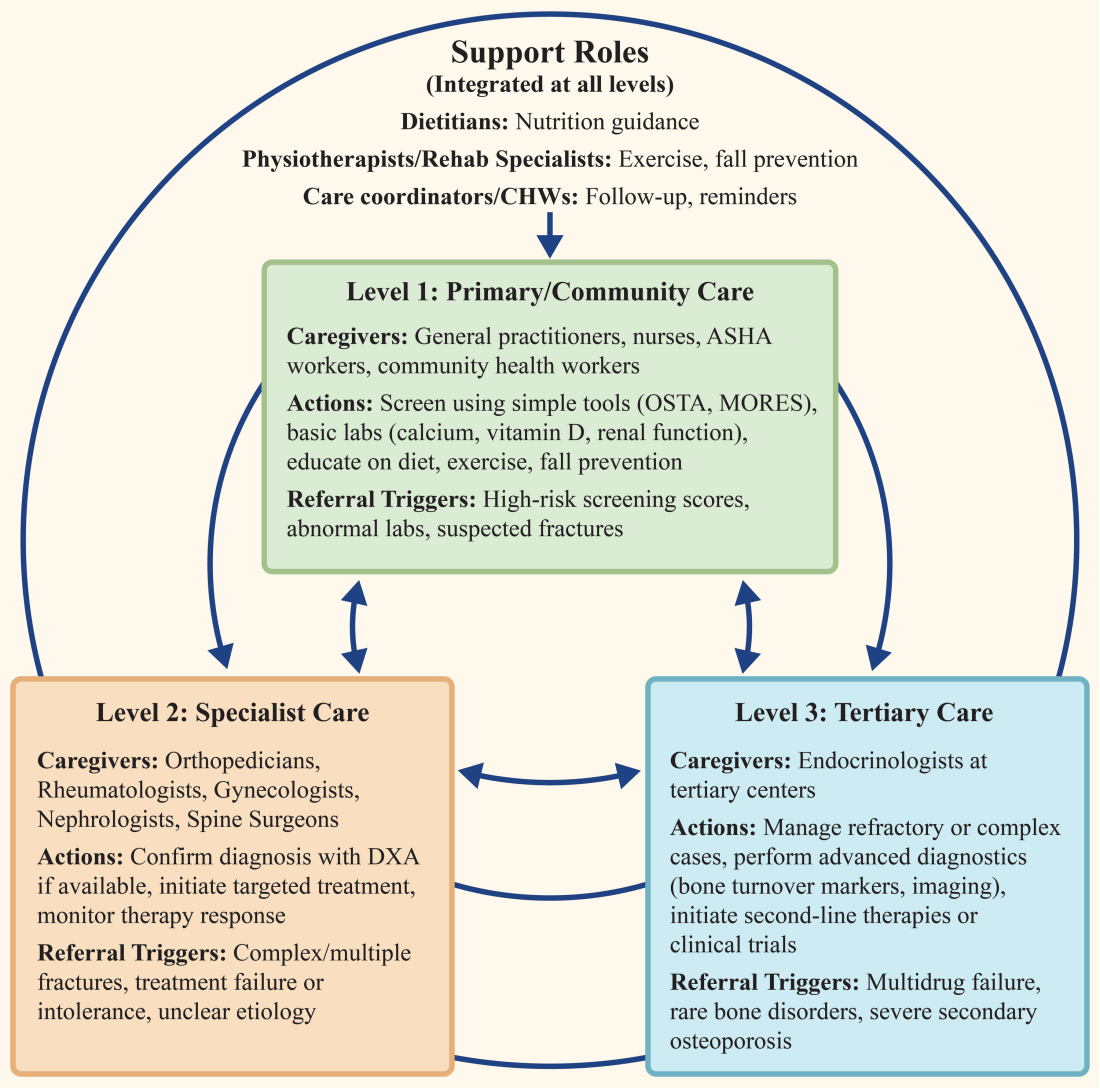
Three-level, resource-sensitive implementation framework for India ASHA: Accredited Social Health Activist, DXA: dual X-ray absorptiometry, MORES: Male Osteoporosis Risk Estimation Score, OSTA: Osteoporosis Self-Assessment Tool for Asians Image Credit: Authors using Adobe Illustrator version 30.0 (Adobe Inc., San Jose, CA)

A structured, tiered, and multidisciplinary approach is crucial for effectively managing osteoporosis in the Indian healthcare system. In resource-limited settings in India, multidisciplinary support can be shared among existing personnel. Nurses and Accredited Social Health Activist workers can deliver patient education, lifestyle advice, and support for adherence. Basic dietary counseling and fall-prevention guidance can be integrated into routine primary care and community programs. As illustrated in Figure 5, the framework outlines the roles of various stakeholders, ranging from primary care screening to specialized management and long-term follow-up.

Key clinical practice pearls are summarized in Table 5.

**Table 5:** Clinical practice pearls BMD: bone mineral density, BTMs: bone turnover markers, CKD: chronic kidney disease, DXA: dual-energy X-ray absorptiometry, FRAX®: Fracture Risk Assessment Tool, FLS: fracture liaison services

Sl. No.	Clinical pearl
	Fracture risk assessment should integrate BMD, FRAX®, and vertebral fracture assessment, with appropriate interpretation of T-scores and Z-scores across populations.
	Secondary causes of osteoporosis should be excluded before therapy initiation, and early osteopenia may warrant intervention in selected high-risk individuals.
	Patients at very high or imminent fracture risk may benefit from early anabolic therapy, with subsequent reassessment using BMD and BTMs to guide treatment sequencing.
	Structured care pathways such as FLS support secondary fracture prevention and continuity of care.
	Screening is recommended in women aged ≥60 years and men aged ≥65 years, with earlier evaluation in high-risk groups including diabetes, CKD, and long-term glucocorticoid exposure.
	FRAX® without BMD may be used in resource-limited settings, with DXA confirmation where feasible, for individuals at elevated risk.
	Treatment adherence should be actively monitored, with early identification and correction of modifiable barriers to optimize long-term outcomes.

Limitations

As a narrative review, this manuscript does not include formal meta-analysis or systematic evidence grading, and conclusions should be interpreted in this context.

## Conclusions

Osteoporosis in India remains under-screened, underdiagnosed, and undertreated, and is characterized by poor adherence. Limited awareness and restricted access to cost-effective screening tools hinder the timely evaluation of individuals at risk. Diagnostic inaccessibility and delayed referrals further impede early recognition and appropriate management. Even when diagnosed, treatment initiation is often inadequate, and long-term adherence remains low due to insufficient education and follow-up. Addressing these gaps requires early screening using validated risk assessment tools, timely initiation of therapy, and consistent monitoring, all of which are supported by multidisciplinary teams and coordinated care pathways. Strengthening public health policies, patient education, and referral systems is crucial to enhancing adherence, reducing the fracture burden, and improving bone health outcomes in India’s aging population. From a policy perspective, two immediate priorities merit attention. First, inclusion of DXA screening costs within the coverage framework of national health insurance schemes would substantially reduce the diagnostic gap in underserved populations. Second, integration of FRAX®-based risk stratification and basic calcium/vitamin D supplementation counseling into the existing infrastructure of primary health centers can be a scalable, low-cost intervention that could meaningfully shift the osteoporosis care paradigm in India.
